# A Novel 6-bp Repeat Unit (6-bp RU) of the 13th Intron Within the Conserved *EPAS1* Gene in Plateau Pika Is Capable of Altering Enhancer Activity

**DOI:** 10.3390/ijms26052163

**Published:** 2025-02-28

**Authors:** Qi Tang, Yuhui Xu, Qingchuan Song, Siqi Cao, Yang Li, Xianyong Lan, Liangzhi Zhang, Chuanying Pan

**Affiliations:** 1 College of Animal Science and Technology, Northwest A&F University, No. 22 Xinong Road, Yangling District, Xianyang 712100, China; tangqi960@163.com (Q.T.); xyh20181018@163.com (Y.X.); songqingchuan@nwafu.edu.cn (Q.S.); caosiqi7747@163.com (S.C.); yangli@nwsuaf.edu.cn (Y.L.); lanxianyong79@126.com (X.L.); 2 Key Laboratory of Adaptation and Evolution of Plateau Biota, Northwest Institute of Plateau Biology, Chinese Academy of Sciences, Xining 810008, China

**Keywords:** plateau pika, *EPAS1* gene, indel, enhancer, transcription factor

## Abstract

The plateau pika (pl-pika), a resilient mammal of the Qinghai-Tibet Plateau, exhibits remarkable adaptations to extreme conditions. This study delves into mutations within the *Endothelial PAS Domain Protein 1* (*EPAS1*) gene, crucial for high-altitude survival. Surprisingly, a novel 6-bp insertion/deletion (indel) mutation in *EPAS1*’s Intron 13, along with an additional repeat unit downstream, was discovered during PCR amplification. Genetic analysis across altitude gradients revealed a correlation between this indel’s frequency and altitude, hinting at its role in altitude adaptation. Fluorescence enzyme assays unveiled enhancer activity within Intron 13, where the deletion of repeat units led to increased activity, indicating potential transcription factor binding. Notably, *GCM1* emerged as a candidate transcription factor binding to the indel site, suggesting its involvement in *EPAS1* regulation. These findings enrich our comprehension of high-altitude adaptation in plateau pikas, shedding light on the intricate interplay between genetic mutations, transcriptional regulation, and environmental pressures in evolutionary biology.

## 1. Introduction

The plateau pika (*Ochotona curzoniae*) (pl-pika), a small mammal native to the Qinghai-Tibet Plateau (QTP), has evolved remarkable adaptations to thrive in extreme high-altitude environments. It exhibits specialized physiological traits, including a reduced hypoxic ventilatory response [1], increased skeletal and cardiac mitochondrial content [2], and enhanced tissue microvascular density [3], which collectively optimize oxygen delivery and energy metabolism under low-oxygen conditions. Additionally, its ability to promote the browning of white adipose tissue without disrupting energy balance enables effective cold stress adaptation [4]. These unique adaptations have made the pl-pika a key model for studying high-altitude survival mechanisms.

The *Endothelial PAS Domain Protein 1 (EPAS1)* gene encodes the HIF-2α transcription factor, which plays a crucial role in high-altitude adaptation [5]. Studies have shown that the expression and genetic variation in EPAS1 are closely related to high-altitude adaptation. In the high-altitude population of Tibet, *EPAS1* is highly expressed and associated with reduced pulmonary vascular reactivity and decreased hemoglobin levels, indicating its important function in high-altitude adaptations [6]. Additionally, *EPAS1* enhances the body’s adaptability to hypoxic environments by regulating pathways related to the cardiovascular [7], metabolic [8], and immune systems [9]. In sheep, *EPAS1* expression is positively correlated with altitude and influences oxygen metabolism [10]. In deer mice, *EPAS1* variants reduce ventilatory sensitivity and carotid body growth under chronic hypoxia at high altitudes [11]. In Tibetan horses, two missense mutations in *EPAS1* increase protein stability, promoting blood circulation and oxygen transport [12]. In pl-pika, a G/A mutation at the 5′ splice site of Intron 14 in the *EPAS1* gene, enhancing EPAS1 protein stability and disrupting circadian rhythm [13]. This stable EPAS1 may provide benefits such as heart protection and reduced risk of high hemoglobin levels, aiding the survival of pl-pika on the QTP. However, research on functional mutations of the *EPAS1* gene in pl-pika remains limited, and the specific mechanisms by which these mutations contribute to high-altitude adaptation are not fully understood.

Based on Liu et al.’s research [13], we aimed to examine the frequency of G/A mutation in a larger population of pl-pika. Surprisingly, we also identified a novel 6-bp insertion/deletion (indel) mutation located in Intron 13, analyzed its genetic parameters in pl-pika populations at different altitudes, and investigated the potential impact on the *EPAS1* gene. This is of great significance in enriching our understanding of the molecular mechanisms underlying the *EPAS1* regulation of pl-pika high-altitude adaptation.

## 2. Results

### 2.1. Detection and Genotyping of Mutations in the Pl-Pika EPAS1 Gene

**Figure 1:**
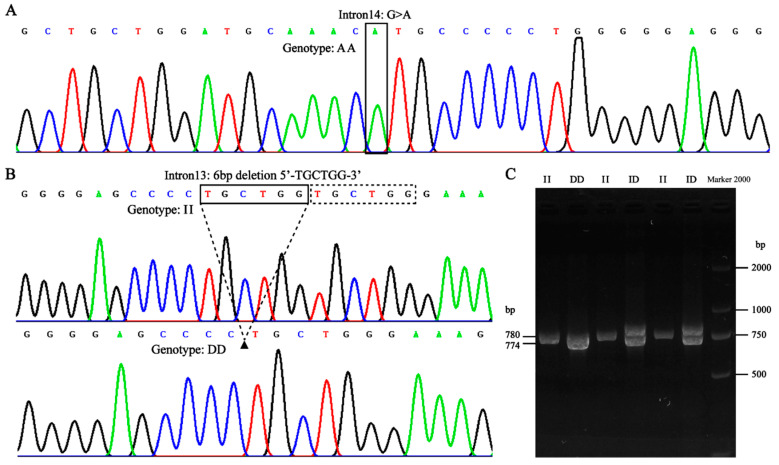
Genotype identification of mutation sites on the pl-pikas *EPAS1* gene: (**A**) The reported G/A mutation at the 5′ splice site of Intron 14 is of the AA genotype in all tested pl-pikas, suggestive of the absence of circadian rhythm. (**B**) Sequencing profile showing a 6-bp deletion mutation in Intron 13 of the pl-pikas *EPAS1* gene. (**C**) Agarose gel electrophoresis reveals the presence of three genotypes (II, ID, and DD) for a 6-bp indel site identified in pl-pikas. Lanes 1, 3, and 5 represent the II genotype, lane 2 represents the DD genotype, lanes 4 and 6 represent the ID genotype, and lane 7 represents the Marker 2000.

To explore the mutation frequency of the G/A mutation site as reported by Liu et al. [13] in pl-pikas, we performed PCR amplification followed by sequencing analysis. Our findings revealed that all tested pikas (*n* = 232) exhibited the AA genotype (Figure 1A and Figure S3), indicating that these pl-pikas do not have a circadian rhythm. Surprisingly, a 6-bp deletion mutation was detected in Intron 13 (Figure 1B and Figure S3). We subsequently identified the genotypes of all individuals using agarose gel electrophoresis, and the results revealed the presence of three genotypes, insertion/insertion (II), insertion/deletion (ID), and deletion/deletion (DD), in pl-pikas (Figure 1C and Figure S4).

### 2.2. Distribution of 6-bp Indel in Pl-Pika EPAS1 Gene at Different Altitude Gradients

**Figure 2:**
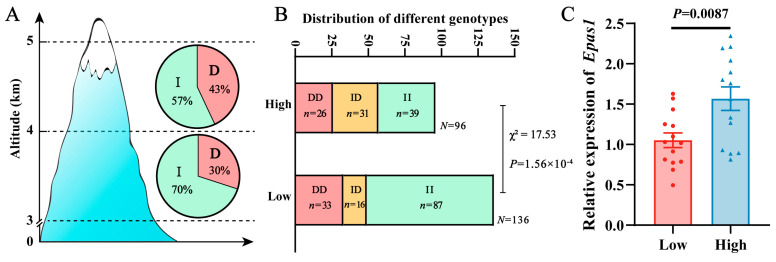
The *EPAS1* 6-bp indel mutation of pl-pikas are differentially distributed between the low-altitude group (3–4 km) and the high-altitude group (4–5 km): (**A**) Distribution of I and D alleles at the 6-bp indel mutation site in high-altitude and low-altitude groups. (**B**) Independent chi-square test showing the differential distribution of genotypes at the 6-bp indel mutation site in high-altitude and low-altitude groups. (**C**) The relative expression levels of the *EPAS1* gene between individuals with the II genotype in the low-altitude group and individuals with the DD genotype in the high-altitude group.

In order to investigate the effect of altitude on the 6-bp indel, we analyzed the distribution of alleles and genotypes across different altitude gradients. The results showed that the frequency of the I allele in the high and low-altitude groups was 57% and 70%, respectively, while the frequency of the D allele was 43% and 30%, respectively. The frequency of the I allele was higher than that of the D allele in both altitude gradients (Figure 2A). Furthermore, the proportions of the II, ID, and DD genotypes in the high-altitude group were 40.63%, 32.29%, and 27.08%, respectively, while those in the low-altitude group were 63.97%, 11.77%, and 24.26%, respectively. An independent chi-square test revealed significant differences in the distribution of genotypes between different altitude gradients (Figure 2B). Compared to the low-altitude group, the high-altitude group had a higher proportion of the D allele, ID and DD genotypes. These findings suggested a potential correlation between the occurrence of the 6-bp indel and altitude in pl-pika populations, with the mutation frequency possibly increasing with an increasing altitude gradient.

To investigate the impact of this indel on the expression of the *EPAS1* gene, the mRNA expression levels of this gene between individuals with the II genotype in the low-altitude group and individuals with the DD genotype in the high-altitude group were analyzed. The results revealed that, in the high-altitude group with a higher mutation frequency, the expression level of the *EPAS1* gene was significantly elevated, approximately 1.6 times higher than that in the low-altitude group (Figure 2C).

### 2.3. The Effect of the 6-bp Repeat Unit Within the EPAS1 Gene on Enhancer Activity

**Figure 3:**
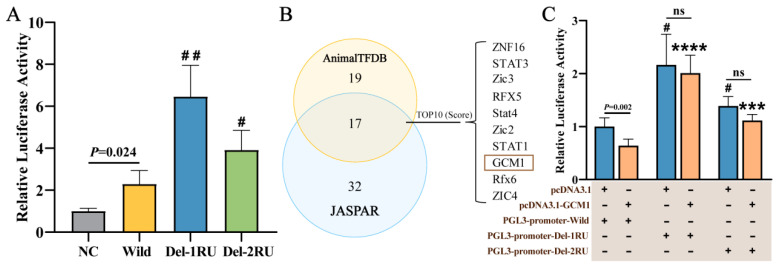
The enhancer activity of the 13th intron of the *EPAS1* gene and the transcription factor binding analysis: (**A**) Dual-luciferase enhancer reporter gene assays are performed on the Wild type and different deletion sequences of the *EPAS1* gene’s 13th intron. # and ## represent *p* < 0.05 and *p* < 0.01, respectively, compared to the Wild group. (**B**) Transcription factors binding to the 6-bp indel site are predicted using Animal TFDB and JASPAR databases. (**C**) The targeted relationship evaluation between *GCM1* transcription factor and different deletion types of the *EPAS1* sequence. # represents *p* < 0.05 compared to the pcDNA3.1 + Wild group; *** and **** represent *p* < 0.001 and *p* < 0.0001, respectively, compared to the pcDNA3.1-GCM1 + Wild group; ns indicates no significant difference (*p* > 0.05).

The repetitive sequence within the intron may be located near the binding sites of enhancer regulatory elements, thereby interfering with the binding of regulatory factors and affecting gene expression levels. Importantly, there is a 6-bp repeat unit present after the 6-bp indel site. To investigate the impact of this repeat unit on enhancer activity, luciferase assays were conducted on three different deletion types: wild, natural mutation (Del-1RU), and engineered mutation (Del-2RU). The experimental results revealed a significant enhancement in fluorescence activity for the Wild group compared to the empty vector control (NC), indicating that the inserted 13th intronic sequence possesses enhancer activity. Both Del-1RU and Del-2RU exhibited a substantial increase in fluorescence activity, with fold changes of 2.81 and 1.71, respectively, relative to the Wild group (Figure 3A). This suggested that this indel mutation can enhance transcriptional activity, with the highest enhancement observed when one repeat unit was deleted.

The increased transcriptional activity caused by the indel mutation may be due to altered accessibility for transcription factors. Therefore, the Animal TFDB and JASPAR online tools were used to predict transcription factors that could bind to the sequence containing the 6-bp indel. The top 10 transcription factors based on their scores were shown in Figure 3B. However, due to the presence of 2RU adjacent to the 6-bp indel (1RU), some transcription factors with high scores were actually binding to 2RU instead of RU1 (e.g., STAT3, Stat4, STAT1). Additionally, based on their functions, *GCM1* and *EPAS1* are both placental regulatory genes and may play roles in hypoxia regulation. Therefore, we identified *GCM1* as a candidate transcription factor. Subsequently, a dual-luciferase assay was conducted, and the results showed that, when different types of reporter vectors were co-transfected with the pcDNA3.1 plasmid, the fluorescence activity followed the same trend as observed with individual transfections. Compared to the pcDNA3.1 + Wild group, the fluorescence activity in the pcDNA3.1-GCM1 + Wild group was significantly reduced, indicating a binding relationship between *EPAS1* and GCM1 (Figure 3C). To further validate whether the binding sites of the two were at the deletion variant site, pcDNA3.1-GCM1 was separately co-transfected with the Del-1RU and Del-2RU reporter vectors. The results showed there was no significant difference between co-transfection of the Del-1RU reporter vector and pcDNA3.1-GCM1 vector, and co-transfection with the pcDNA3.1 vector; the same holds true for Del-2RU (Figure 3C). However, when comparing the fluorescence activity of pcDNA3.1 + Del-1RU and pcDNA3.1 + Del-2RU groups with the pcDNA3.1-GCM1 + Wild group, a significant increase was observed (Figure 3C). This suggests that GCM1 can bind to the 6-bp indel site of *EPAS1*, participating in the regulation of *EPAS1* transcription.

## 3. Discussion

The pl-pika primarily inhabits high mountainous regions at elevations ranging from 3100 to 5300 m and is one of the unique species found in the Qinghai-Tibet Plateau (QTP). Research indicates that pl-pikas at different altitudes show significant variations in adaptive life-history traits, behavioral traits, fat accumulation, and metabolic rates. Pikas at higher altitudes tend to adopt more conservative life history strategies to adapt to harsh environmental conditions, such as shorter growing seasons and limited food resources [14]. Additionally, high-altitude pikas are likely to exhibit more cautious behavioral traits, being more alert and careful about their surroundings [15]. Physiologically, pikas at higher altitudes may accumulate more fat to cope with cold environments and may have higher metabolic rates to maintain body temperature and energy balance [16]. These differences are likely adaptive responses to varying altitude environments, aiding pikas in survival and reproduction. Therefore, we focused on liver and muscle tissues for our study, as the liver is the central organ in metabolism, and muscle tissue is the primary site for energy consumption and physical activity. QTP is recognized as one of the most sensitive areas to global climate change, where temperatures gradually decrease with increasing altitude. Areas above 4 km are considered extreme high-altitude zones characterized by cold and arid climates with sparse vegetation. Research indicates that the annual average temperature in Maduo, Qinghai Province, at an elevation of 4191 m (m), is −4 °C, classified as a high cold grassland climate; while Guide (3663 m) and Guinan (3321 m) have annual average temperatures of −3.7 °C and 2.3 °C, respectively, representing a plateau continental climate [16]. Consequently, we defined the altitude range of 3 km to 4 km as the relatively lower high-altitude group (abbreviated as low-altitude), and the range of 4 km to 5 km as the extreme high-altitude group (abbreviated as high-altitude). This differentiation will facilitate the investigation of genetic variations and expression differences in the *EPAS1* gene in pl-pikas inhabiting high and low altitude environments.

This study is built upon the research conducted by Liu et al. [13] regarding an SNP in *EPAS1*, which, by influencing protein stability, was found to regulate the circadian rhythm and high-altitude adaptation in pl-pikas. To delve deeper into the distribution of this SNP in a larger population, we expanded the scope of our investigation and performed amplification and sequencing of the sequences containing the SNP. The results revealed that all individuals of the pikas tested were homozygous for the mutation, resulting in disrupted circadian rhythms but reduced cardiac damage under hypoxic conditions. Here, we identified a novel 6-bp indel variation in Intron 13 near this SNP, featuring two tandem repeats. Statistical analysis unveiled differential distribution of this indel between high-altitude and low-altitude groups, with a higher proportion of the D allele in high-altitude pikas. This suggests that the D allele may be more advantageous for the high-altitude adaptability of pikas. Furthermore, the results of *EPAS1* gene expression showed that the expression level of DD genotype individuals in the high-altitude group was significantly higher than that of II genotype individuals in the low-altitude group. This finding was consistent with previous reports indicating a positive correlation between *EPAS1* expression and altitude [10,17]. Therefore, we speculated that the 6-bp indel mutation may facilitate the involvement of the *EPAS1* gene in the high-altitude adaptation of pikas. To explore the potential functional regulatory role of this mutation, we conducted luciferase assays, revealing a significant increase in enhancer activity when including the Intron 13 sequence. Moreover, after the deletion of 6-bp and 12-bp, enhancer activity further significantly increased, indicating that this repetitive sequence may serve as a functional enhancer element.

The plateau population mainly adapts to high-altitude environments through genetic mutations, alterations in hemoglobin structure and function, and other mechanisms [18]. Numerous studies have indicated the crucial role of *EPAS1* in high-altitude hypoxia adaptation. Liu et al. [12] found that *EPAS1* exhibits the strongest positive selection features in the genomes of high-altitude Tibetan horses, with two missense mutations closely associated with hematological parameters and potentially affecting the stability of EPAS1 protein. Additionally, variations in non-coding regions also modulate adaptation to high-altitude environments. Association analysis revealed that an SNP in the 3′UTR region of the Tibetan sheep *EPAS1* gene is associated with increased mean red blood cell hemoglobin concentration and mean red blood cell volume [19]. Furthermore, intronic SNPs of the human *EPAS1* gene exhibit significant differences in enhancer activity under normoxic and hypoxic conditions, as well as between Tibetan and Han populations [20]. Therefore, identifying potential functional mutation sites in the *EPAS1* gene of pikas is of paramount importance for enriching the genetic diversity of *EPAS1* and elucidating its involvement in regulating high-altitude adaptation mechanisms. However, structural variations in genes such as *EGLN1* and *HIF1A* in zokors lead to changes in the expression profile of key genes associated with the physiological response to low oxygen levels, indicating that the high-altitude adaptation in rodents involves the combined action of multiple key genes. Therefore, the further exploration of additional genes is warranted [21]. Furthermore, the sequence flanking the 6-bp indel identified in the pl-pika demonstrates limited homology with corresponding regions in other species. This species-specific mutation may be related to the pika’s adaptation to high-altitude environments, potentially reflecting genetic changes that confer survival advantages in response to the unique ecological pressures of such environments.

Given the well-established regulatory role of transcription factors in modulating enhancer activity [22], we identified *GCM1* as a candidate for further investigation. *GCM1* was originally discovered in the nervous system of fruit flies, and its mutation leads to the disappearance of outer layer glial cells [23]. In mammals, *GCM1* expression is predominantly concentrated in the labyrinth layer cells of placental tissue, suggesting a potentially important role in both embryonic development and placental function. Studies have indicated that GCM1 expression decreases under hypoxic conditions, accompanied by the inhibition of syncytiotrophoblast formation [24]. Additionally, disruption of the PHD2 domain protein of HIF-α leads to severe embryonic developmental issues, including placental defects, resulting in the reduced expression of the placental labyrinth marker GCM1 [25]. In some studies, GCM1 has been reported to activate transcription [26], but other studies suggest that GCM1 can decrease transcriptional activity [27]. For example, the antagonistic function between GCM1 and ΔNp63α regulates trophoblast induction efficiency. The dual role of GCM1 may be attributed to its interactions with different co-regulators or binding partners, as well as changes in intracellular and extracellular environments. In our study, we found that the indel mutation may regulate enhancer activity by altering transcription factor binding. In the Wild type, *GCM1* binds to the 6-bp sequence to suppress enhancer activity, whereas, in Del-1RU and Del-2RU, the partial or complete deletion of the 6-bp sequence prevents *GCM1* binding, thereby releasing suppression and increasing enhancer activity. However, the lower activity of Del-2RU compared to Del-1RU may be due to the exposure of new inhibitory transcription factor binding sites or the disruption of functional elements in the core enhancer region caused by the deletion of two repeat units. This discovery provides crucial insights for further deciphering the functional aspects of the *EPAS1* gene and its role in high-altitude adaptation. However, this study has some limitations. For example, we only used the dual-luciferase reporter assay, which reflects the interaction between transcription factors and target genes indirectly through changes in the reporter gene expression, rather than employing other methods such as EMSA and/or RIP assays to directly verify this binding. In future research, we plan to collect fresh tissues of pl-pika to obtain high-quality protein samples and conduct additional experiments to further validate our findings. Furthermore, it is essential to validate other candidate transcription factors. Subsequent studies should consider screening and analyzing additional candidate transcription factors to gain a comprehensive understanding of the complexity and regulatory mechanisms of the transcription factor-target gene network. Collectively, the regulatory relationship in the expression levels between *EPAS1* and GCM1 strengthens their potential association in high-altitude adaptation, offering new perspectives for understanding the molecular mechanisms of these genes in high-altitude adaptability.

## 4. Materials and Methods

### 4.1. Samples and Data Collection

A total of 232 healthy adult pl-pikas, with a consistent male-to-female ratio (Figure S1), were captured using the live-trapping method (Figure S2) and placed in disinfected cages by members of the Northwest Plateau Institute of Biology. Detailed capture locations and sample size are shown in Table 1. Based on geographic, climatic, and ecological factors of the pl-pika’s habitat, altitudes of 3–4 km (km) were classified as the relatively lower habitat altitudes (abbreviated as low-altitude), while altitudes of 4–5 km were classified as the relatively higher habitat altitudes (abbreviated as high-altitude) (Note: This naming convention is used exclusively within the scope of this study to avoid confusion with general altitude classifications). After being transported to the laboratory, the captured pikas were anesthetized using isoflurane (Hengfengqiang Biotech Co., Ltd., Nantong, China), and, approximately 5 min (min) later, they were humanely euthanized by cervical dislocation. Tissue samples were then collected and stored at −80 °C for subsequent experiments.

### 4.2. DNA Extraction

Genomic DNA was extracted from the liver or muscle tissues of pl-pikas using the phenol-chloroform method. Briefly, frozen tissue samples were finely chopped with sterile scissors and digested in lysis buffer containing proteinase K at 65 °C. DNA was then extracted using Tris-saturated phenol and chloroform, followed by precipitation with absolute ethanol. The DNA pellet was washed with 70% ethanol, air-dried, and resuspended in ddH_2_O. DNA concentration and purity were assessed using a Nanodrop 2000 UV-Vis spectrophotometer (Thermo Scientific, Waltham, MA, USA). Samples with A260/A280 ratios of 1.8–2.0 and A260/A230 ratios ≥ 2.0 were considered high-quality and diluted to a working concentration of 50 ng/μL for subsequent analysis.

### 4.3. Primer Designing, Polymerase Chain Reaction (PCR), and Sequencing

The amplification primers targeting the G/A mutation site were designed using the NCBI Primer-BLAST online tool (https://www.ncbi.nlm.nih.gov/tools/primer-blast/, accessed on 18 December 2022). The forward primer 5′-CTCCCCAACAAGCTCATGGT-3′ and reverse primer 5′-GGTGCTTGGAGATGCCTGAT-3′ were synthesized by Tsingke Biotech Co., Ltd. (Xi’an, China). The resulting PCR product has a length of 780 bp.

The PCR system had a total volume of 13 μL, including 6.5 μL 2 × Taq PCR Master Mix (BBI Life Sciences, Shanghai, China), 0.5 μL of both forward and reverse primers, 0.5 μL of diluted DNA, and 5 μL of ddH_2_O. Subsequently, the Touch-Down PCR program was performed on a Genesy 96T thermal cycler (Tianlong Tech. Co., Ltd., Xi’an, China), with a denaturation step at 95 °C for 5 min, followed by 18 cycles of regular PCR, consisting of denaturation at 94 °C for 30 s, annealing at 68 °C for 20 s, and extension at 72 °C for 50 s, with a decrement of 1 °C in annealing temperature per cycle. Then, 30 cycles of regular PCR were carried out at an annealing temperature of 50 °C, followed by a final extension at 72 °C for 10 min.

The PCR products were analyzed by 3.5% agarose gel electrophoresis using 5 μL of the sample, with Marker 2000 (Tsingke Biotech Co., Ltd., Xi’an, China) as the DNA size standard. For Sanger sequencing to determine the genotype, the remaining PCR products were pooled for every 10 samples and sent to Sangon Biotech Co., Ltd. (Shanghai, China).

### 4.4. Transient Transfection and Luciferase Assay

The 13th intron of the *EPAS1* gene contains a 6-bp indel and a 6-bp tandem repeat. The 182-bp sequence from Intron 13, along with sequences of 176-bp and 170-bp obtained after deletion of 6-bp (deletion 1 repeat unit (RU)) and 12-bp (deletion 2 RU), respectively, were cloned into the pGL3-promoter vector. These constructs were designated as Wild, del-1RU, and del-2RU, respectively. The *Glial Cells Missing 1* (*GCM1*) gene’s CDS region from pl-pika was cloned into the pcDNA3.1(+) vector. HEK 293T cells were cultured in DMEM medium supplemented with 12% fetal bovine serum and 1% penicillin/streptomycin, were seeded into a 96-well plate at a density of 1.5 × 10^4^ cells/well, and then transfected using Hieff TransTM Liposomal Transfection Reagent (Yeasen Biotech Co., Ltd., Shanghai, China) as per the manufacturer’s instructions, along with PRL-TK used as an internal control. The blank group (non-transfected) was also included to assess transfection efficiency. After 24 h, luciferase activity was measured using the dual-luciferase Reporter Assay System (Yeasen Biotech Co., Ltd., Shanghai, China) on a SuPerMax 3100 Labsystems Multiskan (Shanpu, Shanghai, China) [28]. All experiments were performed in quadruplicate and repeated independently at least three times.

### 4.5. Transcription Factor Prediction

Transcription factors were predicted using AnimalTFDB v4.0 (http://bioinfo.life.hust.edu.cn/AnimalTFDB4/, accessed on 15 March 2023), where the Intron 13 sequence was input to identify transcription factors binding to mutation sites with a Q-value < 0.01. Similarly, transcription factors for vertebrates were predicted using JASPAR 2022 (https://jaspar.genereg.net/, accessed on 15 March 2023), and those with a Score > 5 were selected.

### 4.6. RNA Extraction and Quantitative Real-Time PCR (qRT-PCR)

Total RNA from liver tissue was extracted using the TRIzol method, and, then, 1000 ng of RNA was reverse transcribed into cDNA using the Hifair^®^III 1st Strand cDNA Synthesis Kit (Yeasen Biotech Co., Ltd., Shanghai, China). Subsequent qRT-PCR was performed on the ABI QuantStudio 5 real-time PCR instrument, with a reaction system consisting of 6 μL of SYBR qPCR Master Mix (Vazyme Biotech Co., Ltd., Nanjing, China), 1 μL each of upstream and downstream primers (10 μM), and 4 μL of 50 × diluted cDNA. The qRT-PCR primers used for detecting the expression level of the *EPAS1* and *GAPDH* gene were as follows: For *EPAS1*, the forward primer sequence was 5′-ACGGAGCGGGACTTTTTCAT-3′, and the reverse primer sequence was 5′-TATGGCGGCTCAGGAAAGTC-3′. For *GAPDH*, the forward primer sequence was 5′-AGGGCTGCTTTCAACTCTGG-3′, and the reverse primer sequence was 5′-CCGTTCTCAGCCTTCACTGT-3′.

### 4.7. Data Statistics and Analysis

To analyze the genotype distribution of the 6-bp indel site, an independent chi-square test was performed using SPSS software (version 24.0; IBM Corp., Armonk, NY, USA) to examine the differences in genotype frequencies at various altitudes. For the luciferase assay and qRT-PCR, *p*-values were calculated using the Student’s *t*-test. *p* < 0.05 was considered statistically significant.

## 5. Conclusions

This study identified a 6-bp indel site in the pl-pika *EPAS1* gene, showing differential distribution in pl-pika populations at different altitudes. Additionally, this site can modulate enhancer activity by regulating the binding of the transcription factor *GCM1*.

## Figures and Tables

**Table 1:** Capturing locations and sample sizes of pl-pika.

Sampling Time	Place	Longitude	Latitude	Elevation	Sample Size
August 2019 and November 2019	Kunlun Mountain Pass	100°18′ E	38°33′ N	4700 m	23
	Maduo County	98°23′ E	34°88′ N	4400 m	13
	Guozhuoyi Mountain Pass	98°60′ E	35°46′ N	4300 m	19
	Gouli Township	98°13′ E	35°75′ N	3900 m	22
	Reshui Township	98°46′ E	36°01′ N	3600 m	21
	Haibei Autonomous Prefecture	100°90′ E	36°95′ N	3300 m	17
	Haibei Autonomous Prefecture	100°90′ E	36°95′ N	3100 m	23
August 2021	Kunlun Mountain Pass	100°18′ E	38°33′ N	4800 m	21
	Maduo County	98°23′ E	34°88′ N	4200 m	19
	Guide County	101°41′ E	36°36′ N	3700 m	19
	Maqin County	100°27′ E	34°45′ N	3700 m	17
	Guinan County	100°66′ E	35°50′ N	3300 m	18

## Data Availability

No new data were created or analyzed in this study.

## Supplementary Materials

The following supporting information can be downloaded at https://www.mdpi.com/article/10.3390/ijms26052163/s1.
