# Supplementary material for: A Novel 6-bp Repeat Unit (6-bp RU) of the 13th Intron Within the Conserved EPAS1 Gene in Plateau Pika Is Capable of Altering Enhancer Activity
Source: Int J Mol Sci. 2025 Feb 28;26(5):2163. doi: 10.3390/ijms26052163 (PMC11901085; doi:10.3390/ijms26052163)
Supplement: Supplementary file 1 [file ijms-26-02163-s001.zip › ijms-3450292-supplementary.pdf]

Supplement Figures

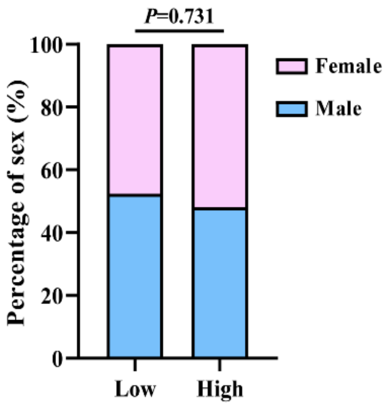

Figure S1 The sex distribution between high-altitude and low-altitude groups (Independent chi-square test).

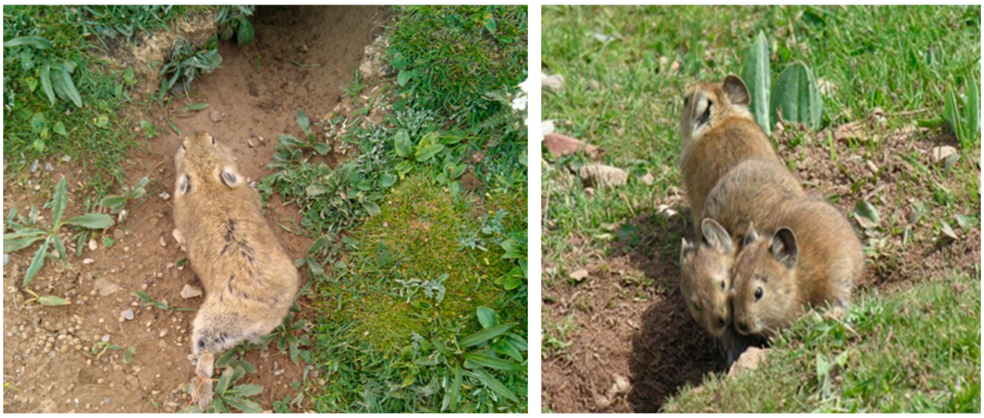

Figure S2 The Pl-pikas captured using the live-capture method.

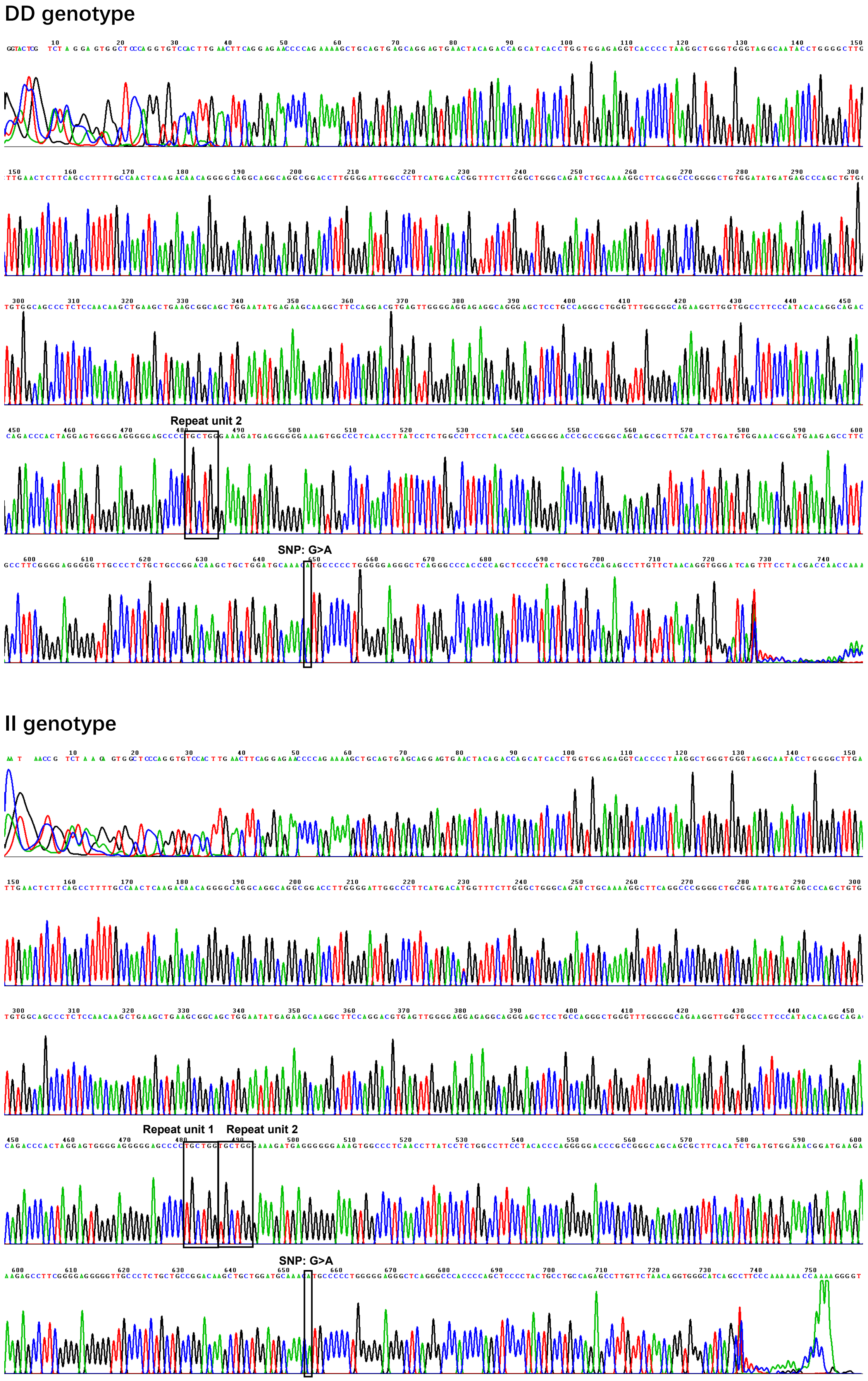

Figure S3 Forward sequencing chromatogram of PCR amplification products.

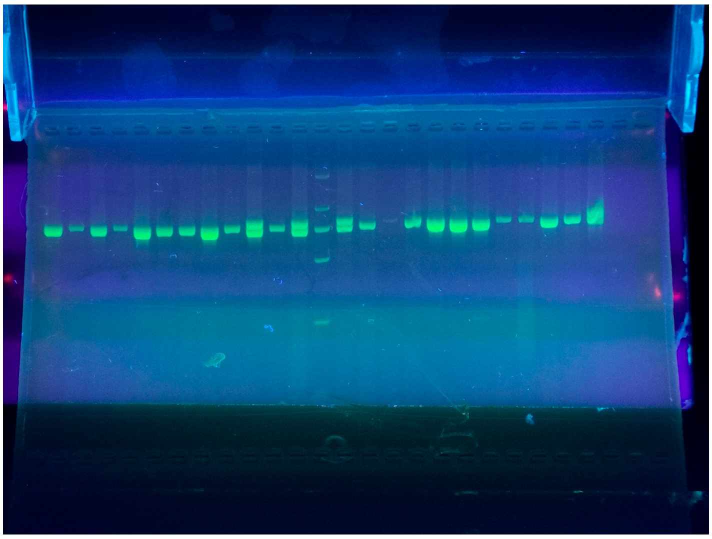

Figure S4 The original electropherograms of Figure 1C.
